# IκBα deficiency in brain leads to elevated basal neuroinflammation and attenuated response following traumatic brain injury: implications for functional recovery

**DOI:** 10.1186/1750-1326-7-47

**Published:** 2012-09-19

**Authors:** Hong Lian, David J Shim, Samson SK Gaddam, Jennifer Rodriguez-Rivera, Brittany R Bitner, Robia G Pautler, Claudia S Robertson, Hui Zheng

**Affiliations:** 1 Huffington Center on Aging Baylor College of Medicine, Houston, TX, 77030, USA; 2 Department of Molecular and Human Genetics, Baylor College of Medicine, Houston, USA; 3 Department of Neuroscience and Medical Scientist Training Program, Baylor College of Medicine, Houston, USA; 4 Department of Neurosurgery, Baylor College of Medicine, Houston, USA; 5 Department of Molecular Physiology and Biophysics, Baylor College of Medicine, Houston, USA; 6 Interdepartmental Program of Translational Biology and Molecular Medicine, Baylor College of Medicine, Houston, USA

**Keywords:** NFκB, IκBα, Conditional knockout mice, TBI, Neuroinflammation, Cerebral blood flow, MRI

## Abstract

**Background:**

The transcription factor NFκB is an important mediator of cell survival and inflammation in the immune system. In the central nervous system (CNS), NFκB signaling has been implicated in regulating neuronal survival following acute pathologic damage such as traumatic brain injury (TBI) and stroke. NFκB is normally bound by the principal inhibitory protein, IκBα, and sequestered in the cytoplasm. Activation of NFκB requires the degradation of IκBα, thereby freeing NFκB to translocate to the nucleus and activate the target genes. Mice deficient in IκBα display deregulated and sustained NFκB activation and early postnatal lethality, highlighting a critical role of IκBα in NFκB regulation.

**Results:**

We investigated the role of IκBα in regulating NFκB activity in the brain and the effects of the NFκB/IκBα pathway in mediating neuroinflammation under both physiological and brain injury conditions. We report that astrocytes, but not neurons, exhibit prominent NFκB activity, and that basal NFκB activity in astrocytes is elevated in the absence of IκBα. By generating mice with brain-specific deletion of IκBα, we show that IκBα deficiency does not compromise normal brain development. However, basal neuroinflammation detected by GFAP and Iba1 immunoreactivity is elevated. This leads to impaired inflammatory responses following TBI and worsened brain damage including higher blood brain barrier permeability, increased injury volumes and enlarged ventricle volumes.

**Conclusions:**

We conclude that, in the CNS, astrocyte is the primary cell type subject to NFκB regulation. We further demonstrate that IκBα plays an important role in regulating NFκB activity in the brain and a robust NFκB/IκBα-mediated neuroinflammatory response immediately following TBI is beneficial.

## Background

Traumatic brain injury (TBI) is a major cause of mortality and disability in young adults and is particularly prevalent among combat veterans of recent conflicts. Besides the acute brain damage, TBI is associated with increased risk of dementia later in life (reviewed in [1,2]). The pathophysiology of TBI is highly heterogeneous and complex. Following the primary mechanical insults, TBI induces a multifactorial tissue response, including the breakdown of blood–brain barrier (BBB) and activation of inflammatory pathways, within hours to days following the trauma, and these have been shown to influence the neuronal survival and functional recovery (reviewed in [3]). Neuroinflammation is a prominent feature associated with TBI. In particular, TBI triggers the activation of astrocytes and microglia and the release of proinflammatory cytokines including TNFα, IL-1β and IL-6 (reviewed in [4,5]). However, the functional consequences of the neuroinflammatory response remain controversial. In mouse and rat models of TBI, some studies show that the cytokine release promotes BBB breakdown and worsens brain damage and functional outcome [6-10], while other reports suggest that some aspects of inflammation may be beneficial to the brain [4,11-14].

The transcription factor nuclear factor kappa B (NFκB) is a master regulator of inflammation. It also mediates a variety of other cellular processes including cell survival and apoptosis (reviewed in [15]). NFκB activity is tightly regulated. It is normally bound by the principal inhibitory protein, IκBα, and is sequestered in the cytoplasm. NFκB can be activated by cytokines or other stimuli including trauma. This requires the degradation of IκBα through the ubiquitin proteasome system, thereby freeing NFκB to translocate to the nucleus and activate the target genes containing a consensus κB site in their promoters [16,17], one of which is IκBα [18]. This forms an effective feedback inhibitory loop, leading to the silencing of NFκB. Accordingly, mice deficient in IκBα display deregulated and sustained NFκB activation and early postnatal lethality [19,20], highlighting a critical role of IκBα in NFκB regulation. NFκB activity is elevated immediately following brain injury and sustained for a long period of time afterwards [21-23]. Both neuroprotective and neurotoxic roles of the NFκB pathway have been proposed. One possible reason for these dual effects of inflammation is that NFκB activation may induce expression of different pathways in the brain. Another possibility is that the balance between the beneficial and detrimental effects of inflammation may be determined by the timing and intensity of the response [15,24,25]. A better understanding of the complex inflammatory response to brain injury may lead to more effective treatment of TBI.

To bypass the lethality and to investigate the function of the NFκB/IκBα pathway in adult brain, we generated brain-specific IκBα conditional knockout mice. We show that IκBα deficiency leads to elevated basal neuroinflammation, resulting in a failure to mount proper inflammatory responses following TBI and worsened brain damage.

## Results

### Deletion of IκBα in the brain leads to upregulated astroglia NFκB activity and neuroinflammation

**Figure 1:**
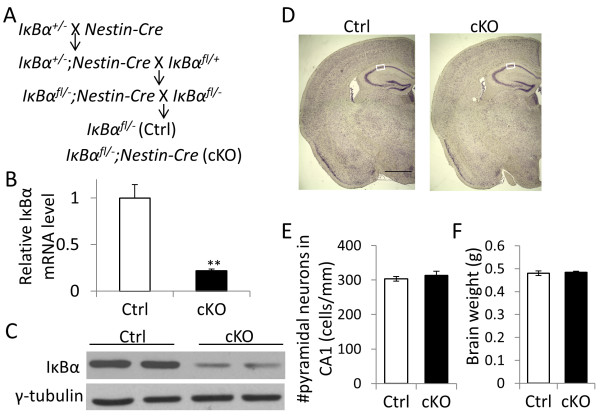
**Generation and characterization of brain IκBα conditional knockout (cKO) mice.** (**A**) Breeding scheme used for generating the IκBα cKO mice and the littermate controls (Ctrl). (**B**) Real-time quantitative PCR (qPCR) analysis of IκBα mRNA (N = 3/genotype) and (**C**) Western blot analysis of IκBα protein levels in brain lysates of 2–3 month old adult cKO and Ctrl hippocampal tissue. (**D**) Representative images of Nissl-stained brain sections of 10–12 month old Ctrl and cKO mice. Scale bar: 1 mm. White boxes highlight the sampling region for counting pyramidal neuronal number. (**E**) Quantification of pyramidal neurons in hippocampal CA1 region (N = 5/genotype) and (**F**) brain weight measurements of Ctrl and IκBα cKO mice (N = 9-14/genotype).

We generated mice with specific deletion of IκBα in the brain (IκBα cKO) by crossing an *IκBα* floxed allele [26] with the *Nestin-Cre*[27] transgenic mice (Figure 1A). Littermate IκBα^fl/-^ mice without Cre transgene was used as controls (Ctrl). Quantitative real-time PCR (qPCR) analysis showed that the IκBα mRNA level was significantly reduced (Figure 1B). Immunoblotting of IκBα revealed a similar level of reduction in protein expression in the cKO mice (Figure 1C). In contrast to the IκBα germline knockout mice, which are early postnatal lethal, the IκBα cKO mice are viable and overtly normal. Nissl staining of adult IκBα cKO brains revealed indistinguishable morphology compared to the littermate controls (Figure 1D). Further quantification of pyramidal neurons in CA1 region showed no overt neurodegeneration in the IκBα cKO mice (Figure 1E) and this is in agreement with their normal brain weight (Figure 1F).

**Figure 2:**
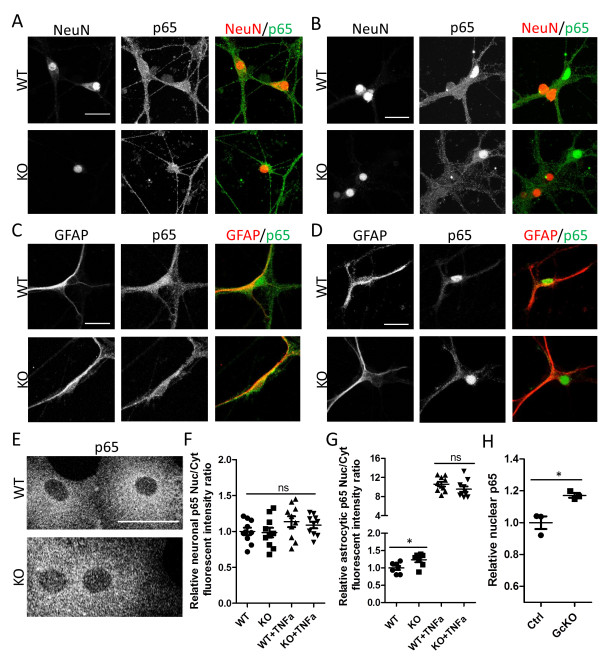
**IκBα deletion results in increased NFκB activity in astrocytes.** (**A** and **B**) Representative images of wild-type (WT) and IκBα knockout (KO) primary neurons, either under basal condition (**A**) or treated with 50 ng/ml TNFα for 30 min (**B**), followed by staining against neuronal marker Neuronal nuclei (NeuN) and NFκB subunit p65, and displayed as individual or merged images. Note the uniform p65 staining in NeuN-positive neurons under both conditions and the cells that undergo p65 nuclear translocation in response to TNFα (**B**) are NeuN-negative. (**C** and **D**) Representative images of primary astrocytes, either under basal condition (**C**) or treated with TNFα (**D**), followed by staining against astroglia marker GFAP and p65, and displayed as individual or merged images. Note clear p65 nuclear translocation in GFAP-positive astrocytes. (**E**) Representative p65 immunostaining of primary WT and KO astroglia cultures. (**F**) Quantification of relative p65 nuclear to cytoplasmic intensity in WT and IκBα KO neurons with or without TNFα. (**G**) Quantification of relative p65 nuclear versus cytoplasmic fluorescence intensity in WT and IκBα KO astrocytes in the presence or absence of TNFα stimulation, documenting increased basal p65 in IκBα KO sample and greatly enhanced nuclear p65 upon TNFα treatment in both WT and KO cultures. N = 7-10/genotype. (**H**) p65 ELISA quantification of nuclear preparations from adult (2–3 month) Ctrl and IκBα astroglia-specific knockout (GcKO) hippocampal samples. N = 3/genotype. ns: non-significant; *p < 0.05. Scale bar: 25 μm.

As a predominant inhibitor and downstream target of NFκB, IκBα plays an essential role in regulating NFκB activity. Both activation and suppression of NFκB by IκBα have been reported, likely mediated in a cell type-specific manner [19,20,26]. Accordingly, we prepared primary neuronal and primary astrocyte cultures from the germline IκBα knockout neonates and performed immunostaining using an antibody that recognizes the major NFκB subunit, p65 (Figure 2). Neurons from wild-type (WT) and IκBα knockout (KO) mice showed similar weak and homogeneous p65 distribution over the whole cell body (Figure 2A), indicative of non-specific staining. This is corroborated by the similar staining pattern following treatment with the potent NFκB activator TNFα (Figure 2, B and quantified in F). Of interest, the cells that show clear p65 nuclear translocation in response to TNFα are NeuN-negative, likely due to the contamination of glial cells in the culture (Figure 2B). In contrast, strong cytoplasmic and weak nuclear staining can be detected in GFAP-positive astrocytes using the anti-p65 antibody (Figure 2C). The specificity of the signal was further confirmed by the robust nuclear translocation of p65 upon treating the cultures with TNFα (Figure 2D). Measurement of nuclear and cytoplasmic fluorescence intensity from the control and IκBα KO astrocytes under basal condition revealed higher nuclear to cytoplasmic ratio (Nuc/Cyt) in the absence of IκBα (Figure 2G, WT vs. KO), suggesting that deleting IκBα leads to higher basal NFκB activity. TNFα treatment led to strong nuclear translocation of TNFα. However, p65 Nuc/Cyt ratio after TNFα treatment showed similar values between WT and KO astrocytes likely caused by oversaturated nuclear signal intensity (Figure 2G, WT+ TNFα vs. KO + TNFα). The enhanced basal nuclear NFκB in KO astrocytes is further strengthened by quantifying the nuclear NFκB levels in IκBα conditional knockout mice in which IκBα is specifically deleted in astrocytes by crossing the *IκBα* floxed allele with the *GFAP-Cre* transgenic mice [28] (Figure 2H). Overall, our results provide strong support for the notion that, in the CNS, astrocyte is the primary cell type subject to NFκB regulation and that loss of IκBα in astrocytes results in higher basal NFκB activity. Nevertheless, we cannot exclude the possibility that neuronal NFkB may be induced under pathological conditions *in vivo*.

**Figure 3:**
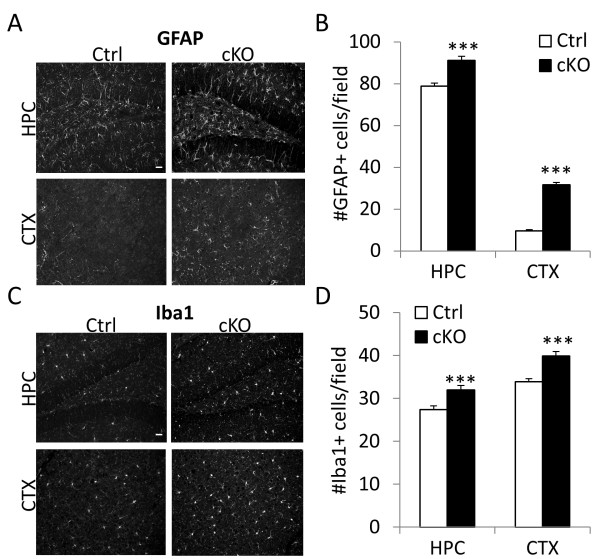
**Elevated neuroinflammation in IκBα cKO mice.** (**A** and **C**) Representative images of brain sections from 10–15 month-old mice Ctrl and IκBα cKO mice stained against astroglia marker GFAP and microglia marker Iba1. HPC: hippocampus. CTX: cortex. (**B** and **D**) Quantification of GFAP- and Iba1- positive cells in HPC and CTX respectively. N = 4/genotype. For each mouse, 3 evenly spaced sections were stained and quantified. ***p < 0.001. Scale bar: 20 μm.

Considering that NFκB is a master regulator of inflammatory responses, we tested the levels of neuroinflammation in IκBα cKO mice by immunostaining the brain sections with antibodies against GFAP and Iba1 and quantifying the number of astrocytes and microglia respectively (Figure 3). Indeed, the number of GFAP- and Iba1-positive cells in both the hippocampus (HPC) and cortex (CTX) are significantly increased in IκBα cKO brains as compared to their littermate controls (Figure 3B and D).

### Attenuated neuroinflammatory response in IκBα deficient brain following TBI

**Figure 4:**
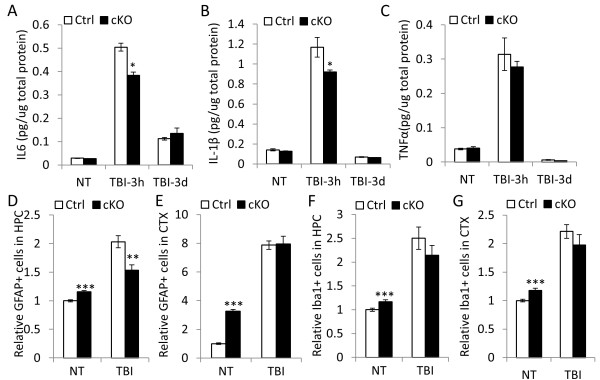
**IκBα inactivation in the brain leads to blunted neuroinflammatory response following traumatic brain injury (TBI).** TBI were performed on 10–12 month old Ctrl and cKO mice. (**A**-**C**) IL6 (**B**), IL-1β (**C**) and TNFα (**D**) levels assayed by ELISA 3 hours and 3 days post-injury. N = 3/genotype. (**D**-**G**) Relative GFAP-positive (**D** and **E**) or Iba1-positive (**F** and **G**) cells in HPC (**D** and **F**) or CTX (**E** and **G**) in response to TBI at 14 days after TBI. All values were normalized to numbers of NT Ctrl mice. N = 6 mice/genotype. For each mouse, 3 evenly spaced sections were used for quantification. *p < 0.05; **p < 0.01; ***p < 0.001.

Neuroinflammation is an invariable feature associated with TBI. This involves the activation of NFκB and the release of pro-inflammatory cytokines including TNFα, IL-1β and IL-6 by astrocytes and microglia. Having characterized the IκBα cKO mice under basal condition, we were interested in understanding how the basal activation of NFκB affects the brain damage following TBI. We performed controlled cortical impact (CCI), which is the most-widely used protocol and known to induce moderate to severe brain injury, to Ctrl and IκBα cKO mice, and measured cytokine levels without injury (NT) or 3 hours and 3 days after CCI (Figure 4A-C). As expected, levels of IL6, IL-1β and TNFα protein displayed time-dependent kinetic changes. All cytokines were strongly upregulated in response to the injury shortly after TBI (3 hour) but returned close to baseline 3 days post injury (Figure 4A-C). Noticeably, at 3 hour after injury, CCI-induced IL6 and IL-1β overexpression was significantly lower in the absence of IκBα (Figure 4A and B). This is also the case with TNFα although to a lesser degree (Figure 4C). No group differences were observed for cytokine expression either under basal condition or 3 days post injury. Combined together, these results indicate that the instant cytokine-associated inflammatory response to brain injury in IκBα cKO mice was blunted while later-phase cytokine expression was largely unaffected.

As expected, CCI resulted in prominent increase in reactive astrocytosis and microgliosis in both the control and IκBα cKO mice, quantified by GFAP- and Iba1-immunoreactivity in both hippocampus and cortex (Figure 4D-G). However and in contrast to the basal glia activation in the IκBα cKO brains, the numbers of reactive astrocytes and microglia were overall similar between the two groups with the IκBα cKO mice showing trends of reduction. In fact, the level of astrocytosis was significantly reduced in the hippocampus of the cKO mice compared to the controls (Figure 4D). These lead to an overall reduced degree of neuroinflammation in response to TBI in the IκBα cKO mice, a notion that is in agreement with their reduced cytokine release.

### Loss of IκBα in the brain is associated with worsened tissue damage following TBI

**Figure 5:**
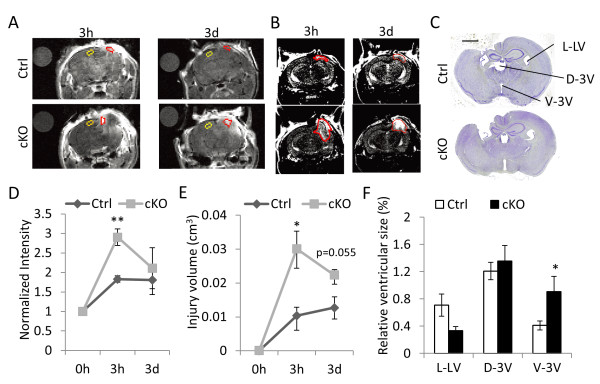
**IκBα cKO mice display increased BBB permeability and tissue damage after TBI.** (**A**) Representative T1-weighted MRI images of Ctrl and cKO mice 3 hour and 3 day after TBI. Water droplets (separate circles in upper left) serve as the background control and uninjured areas (marked in yellow) are used as the internal control. Injured areas were marked in red. (**B**) Representative T2-weighted MRI images of Ctrl and cKO mice 3 hour and 3 day after TBI with the injured brain tissue marked in red. (**C**) Representative images of Nissl stained brain sections from mice survived 14 days after TBI. Scale bar: 1 mm. (**D**) Quantification of relative BBB permeability (normalized intensity) based on T1-weighted MRI images shown in (A). N = 3 for each group. (**E**) Quantification of total injury volume based on T2-weighted MRI images shown in (**B**). N = 3 for each group/time point. (**F**) Relative ventricular sizes were calculated as pixel numbers taken by ventricles divided by that of whole brain substances based on Nissl staining results. L-LV: left lateral ventricle, D-3 V: dorsal third ventricle, V-3 V: ventral third ventricle. N = 5-6 mice/genotype. *p < 0.05; **p < 0.01.

To test the functional effect of attenuated cytokine release resulting from brain IκBα deficiency, we performed MRI scans to measure blood brain barrier (BBB) permeability (Figure 5A) and tissue damage (Figure 5B) in control and IκBα cKO mice at 3 hours and 3 days post-TBI. Quantification of MRI images showed that both the BBB permeability (Figure 5D) and the volume of injured tissue (Figure 5E) exhibited similar time-dependent differences as the cytokine profiles, i.e., significantly higher BBB permeability and brain injury can be detected in IκBα cKO mutant at 3 hours but not 3 days after TBI. Altered cytokine expression and BBB permeability in IκBα cKO mice immediately following TBI is associated with their impaired long-term recovery. Assessment of morphological changes by Nissl staining of comparable sections and quantification of the sizes of left lateral ventricle (L-LV), dorsal third ventricle (D-LV) and ventral third ventricle (V-3 V) 14 days after TBI showed that, while L-LV and D-LV did not show statistical difference, V-3 V was significantly enlarged in IκBα cKO mice (Figure 5C and quantified in F).

## Discussion

In the current study, we investigated the role of IκBα in regulating brain NFκB activity and the output of the NFκB/IκBα pathway in mediating neuroinflammation in both basal and brain injury conditions. Using neuronal and astrocytic cultures isolated from wild-type and IκBα knockout mice, we show here that astrocytes, but not neurons, exhibit prominent NFκB activity, and that NFκB levels in astrocytes are elevated in the absence of IκBα. IκBα deficiency in the brain does not compromise normal brain development. However, basal neuroinflammation detected by GFAP and Iba1 immunoreactivity is increased. This leads to impaired inflammatory responses following TBI and worsened brain damage including higher blood brain barrier permeability and larger injury volumes and enlarged ventricles in the brain.

NFκB has been shown to play important roles in normal brain development and neuronal survival and function [29-33]. This has been attributed, at least in part, by the presence of constitutive NFκB activity in the neurons and its further induction in response to multiple stimuli [34-38]. However, this notion was counted by a report showing that neuronal NFkB was not responsive to a variety of stimuli [39]. Importantly, a careful analysis and comparison of an extensive list of NFκB antibodies by Herkenham et al. documented that many of the antibodies used to detect neuronal NFκB were not specific to the target proteins and that the antibodies proven to be specific to NFκB subunits failed to detect any constitutive NFkB above background in neurons [40]. Our results that neurons exhibit minimum levels of NFκB and that it does not respond to TNFα treatment are consistent with these recent studies. Therefore, it is possible that the neuronal phenotypes observed in various NFκB mutant animals are originated from glia cells and through neural-glia interactions. However, we cannot exclude the possibility that neuronal NFkB may be induced under pathological conditions *in vivo*.

In light of the critical role of IκBα in NFκB regulation, the small (10-15%) increase in basal NFκB activity in IκBα deficient samples is somewhat unexpected. Nevertheless, this subtle alteration is consistent with a previous report which measured NFκB activities in different tissues and cell types of germline IκBα knockout mice, the results of which revealed that the regulation of NFκB by IκBα is cell type-dependent with brain displaying only slightly increased NFκB activity in the absence of IκBα [19]. The studies combined suggest that other IκB proteins such as IκBβ may compensate for the loss of IκBα in the brain, leading to overtly normal brain morphology in CNS-specific IκBα conditional knockout mice. However, the dynamic roles played by IκBα in NFκB regulation are unlikely to be fully replaced by other IκB proteins. For instance, IκBβ or IκBϵ cannot efficiently shuttle between nucleus and cytoplasm like IκBα does [41] and their resynthesis rate after degradation are much slower than IκBα [42]. This may explain the attenuated instant inflammatory responses after TBI observed in the IκBα cKO mice. Following this initial period, nuclear NFκB activity gradually turns off and other IκB proteins may eventually compensate for the inhibitory function of IκBα [19,42]. Our results that after 3 days, cytokine levels and BBB permeability were similar between the control and IκBα cKO mice are consistent with this assessment. Nevertheless, it remains possible that the impaired response in the absence of IκBα may be due to other unmeasured pathways in addition to increased NFkB.

NFκB activity has been shown to be elevated immediately following brain injury and sustained for a long period of time afterwards [21-23]. The activation of NFκB triggers a pleiotropic response involving pathways that both contribute to and protect from brain damage. For example, on the one hand, NFκB activation results in the release of proinflammatory cytokines, and potentially neurotoxic reactive oxygen species and excitotoxins [43,44]. On the other hand, it induces the expression of anti-oxidants and pro-survival factors [45-47]. Activation of NFκB in both residential astrocytes and microglia after brain injury has been reported previously [21,48]. Additionally, traumatic injury leads to compromised BBB which may allow infiltration of peripheral lymphocytes and secretion of cytokines into the brain [49]. Microglia can trigger direct effect in response to TBI [50], it can also undergo modulation by astrocytes [51,52]. The eventual worsened outcome after injury in the mutant mice may be due to the insufficiency of astrocytic activation, and/or ineffective microglial alteration directly or downstream of astrocyte activation. As immunocompetent cells, astrocytes, compared to microglia or invading lymphocytes, displays extensive coverage of brain capillaries [53] and intricate communication with other neural cells. Astrocytes participate in BBB function and are capable to affect BBB permeability [54,55]. The fact that worse BBB permeability is associated with attenuated cytokine expression at 3 hr after CCI may be an evidence for the beneficial role of robust inflammatory responses from astrocytes in functional recovery after brain injury.

Deciphering the precise role of NFκB-mediated neuroinflammation will be beneficial for understanding the potential of NFκB-based therapy. Our results suggest that an initial robust neuroinflammatory response following TBI is beneficial for reducing brain damage. Since the IκBα/NFκB pathway plays an important role in mediating this response, transient activation of NFκB-mediated neuroinflammation immediately after the brain injury may be therapeutically beneficial.

## Conclusions

Traumatic brain injury (TBI) affects a wide range of individuals, from young adults to elderly and from civilians to military combats, and cause both short-term impairments and long-term consequences. The current treatment is limited to symptom management and the mechanisms leading to the short- and long-term effects are poorly understood. A better understanding of the molecular and cellular mechanisms of the various processes accompanying TBI and their relation to functional recovery is critical to identify effective therapies for TBI. Our results demonstrate that a robust NFκB/IκBα-mediated neuroinflammation immediately following TBI is neuroprotective. Therefore, transient stimulation of the neuroinflammatory response through NFκB activation may be therapeutically beneficial.

## Methods

### Animals

Mice were housed 2–5 per cage with *ad libitum* access to food and water in a room with a 12 hr light/dark cycle in a sterile pathogen-free mouse facility. All procedures were performed in accordance with NIH guidelines and with the approval of the Baylor College of Medicine Institutional Animal Care and Use Committee. *IκBα*^*+/−*^ mice [19] and *Nestin-Cre* transgenic mice [27] are available from Jackson Laboratory. Astroglia-specific IκBα knockout mice (IκBα GcKO) were generated using the breeding as the same as that of IκBα cKO mice and the *GFAP-Cre* transgenic mice used were obtained from National Cancer Institute Mouse repository [28]. IκBα floxed mice were obtained from Dr. Rudolf Rupec (University of Munich, Germany) and have been described previously [26]. All animals have been backcrossed onto the C57BL/6 background for a minimum of six generations. Genotyping was performed by PCR of tail DNA at time of weaning.

### Controlled cortical impact (CCI)

Mice at 7–12 month old age were anaesthetized with isoflurane and intubated to control ventilation. After placement on a stereotaxic frame, a 3 mm craniotomy was performed over the right parietal cortex. Injury was induced using a voltage-driven impactor (3 m/sec, 1.5 mm deformation, 100 msec). The wounds were then sutured closed and the mice monitored until full recovery.

### Magnetic resonance imaging (MRI)

Mice survived 3 hour or 3 day after TBI were MRI-scanned to measure time-depended injury volume and blood brain barrier permeability changes. Scans were performed with a 9.4 T Bruker Avance Biospec Spectrometer, 21 cm bore horizontal scanner with a 35 mm volume resonator (Bruker BioSpin) at 3 hrs post-TBI. Mice were initially anesthetized with 5% isoflurane and 100% oxygen and then maintained on 1% to 2% isoflurane during imaging. Respiration and temperature were monitored using a respiratory pad and rectal probe, respectively (Small Animal Instruments). The temperature was maintained at 37°C with an air-heating system. First, after the initial pilot scan, a high resolution, T2-weighted, 3D RARE anatomical scan was performed to allow for the segmentation and measurement of tissue damage volumes. Then, we used dynamic MRI with contrast to assess the degree of BBB permeability. The mice were placed into the magnet along with a water phantom and T1-weighted, 2D multi-slice multi-spin scans were obtained before and 5 min after Magnevist (0.5 mM/kg) injection into the tail vein. Identical series of T1-weighted scans were acquired (10 repetitions) to observe the progression of contrast increase within the tissue. The contralateral side of the brain to the injury served as an internal control. After imaging, mice were allowed to recover on a warmed heating pad prior to being returned to their cage. Images were analyzed using ImageJ software. To calculate size of injury, the area of reactive brain was calculated in each 2D image slice, multiplied by the interslice distance (0.5 mm), and then summed. To calculate BBB permeability, an image slice through the center of injury was selected and ROIs were drawn in the water phantom, uninjured cortex, and injured cortex for each repetition. The mean gray value was then calculated for each ROI and the cortical values were divided by the water value. The maximum normalized intensity was then chosen.

### Primary cell culture and immunocytochemistry

Cortices were isolated from new born pups (P0-P3) in HBSS supplemented with 10 mM HEPES, 0.6% glucose, 1% v/v Pen/Strep under dissecting microscope and cut into small pieces. Cortices was digested with 2.5% trypsin and 1% DNAase I in HBSS at 37°C for 30 min and trypsin inhibitor (1 mg/ml) was then added to stop digestion. Tissue was collected after 1000 rpm for 10 min and triturated and resuspended after centrifugation at 1000 rpm for another 10 min in culture media (For neurons: Neurobasal medium supplemented with 2% B27, 0.5 mM L-glutamine, 0.4% v/v Pen/Strep; For astroglia, DMEM supplemented with 10% bovine calf serum and 1% v/v Pen/Strep). For neuronal culture, cells were plated onto poly-D-lysine (PDL)-coated glass coverslips at 50000 cells/cm^2^ and incubated at 37°C with 5% CO_2_ for 2 weeks before experiments. For astroglia, cells were plated in PDL-coated T-75 flasks at 50000 cells/cm^2^ and when confluent, cultures were shaken at 220 rpm overnight at 37°C to remove unwanted cell types (microglia, neurons and fibroblasts). Pure astroglia cell cultures were then trypsinized with 0.5% trypsin in EDTA and plated onto PDL-coated glass coverslips. Glia cells were used for immunocytochemical experiments after 7–10 days incubation.

Cells were fixed in 4% PFA for 20 min, blocked with 3% goat serum in PBST for 1 hr and incubated in primary antibodies (Rabbit anti-p65, cell signaling, 1:500, Mouse anti-GFAP, Millipore, 1:1000; Mouse anti-NeuN, Millipore, 1:1000) overnight at 4°C, followed by incubation with secondary antibody (Goat anti-Rabbit-Alexa555, Invitrogen, 1:2000; Goat anti-Mouse-Alexa488, Invitrogen, 1:2000) diluted in blocking solution for 2 hr at room temperature. Confocal immunofluorescent images of p65 in primary astroglia were taken using a Leica DM2500B SPE confocal microscope with a 20x objective. Images were analyzed by Adobe Photoshop. Nuclear area was defined by DAPI and quantified for its mean grey value after selection (V_Nuc_). Cytoplasmic intensity was quantified as the mean gray value of a cytoplasmic area with exactly the same size as nuclear area close to the nucleus (V_cyt_). Nuclear/cytoplasmic fluorescent intensity ratio was calculated as V_Nuc_/V_cyt_.

### Quantitative real-time PCR (qRT-PCR)

Total RNA was isolated from snap frozen brain tissues using the RNeasy Mini Lipid kit (Qiagen) and analyzed as described previously [56]. RNA extracted from adult 2–3 month mice were tested for IkBa mRNA level respectively. Samples from 2 ~ 4 mice for each genotype were used for group comparisons. The primer sequences are as follows:

5’-TCGCTCTTGTTGAAATGTGG-3’ (IκBα Fwd)

5’-TCATAGGGCAGCTCATCCTC-3’ (IκBα Rev)

5’-AATGTGTCCGTCGTGGATCTGA-3’ (GAPDH Fwd)

5’-GATGCCTGCTTCACCACCTTCT-3’ (GAPDH Rev)

### Protein isolation, nuclear protein fractionation and western blotting

Hippocampal or cortical tissues were homogenized in RIPA buffer and centrifuged at 14000 rpm for 20 min. Supernatant was collected and quantified using a DC colorimetric protein assay (Bio-Rad). For nuclear protein extraction, brain tissue was homogenized in extraction buffer (10 mM HEPES, 1 mM EDTA, 2 mM EGTA, 1 mM DTT, 0.5 mM PMSF supplemented with protease and phosphatase inhibitors) and centrifuged at 1000xg for 10 min at 4°C to pallet nuclear fraction. Pellet was then rinsed with extraction buffer and resuspended in buffer (20 mM HEPES, 400 mM NaCl, 1 mM EDTA, 1 mM EGTA, 1 mM DTT, 0.5% NP-40 supplemented with protease and phosphatase inhibitors). Lysates were boiled at 95°C for 7 min in sample buffer. 15 μg of protein samples were then loaded onto 12% SDS-polyacrylamide gels, run at 100 V for 2 hr, transferred onto nitrocellulose membranes (Bio-Rad) at 90 V for 1.5 hr at 4°C in transfer buffer (50 mM Tris, 40 mM glycine, 20% methanol, 0.01% SDS), and blocked with 5% milk in Tris-buffered saline containing 0.1% Tween-20 (TBST). The membranes were then probed with primary antibodies (Rabbit anti-IκBα, Santa Cruz, 1:1000; Rabbit anti-p65, Abcam, 1:1000; Mouse anti-γ-tubulin, Sigma, 1:20,000) diluted in blocking solution overnight at 4°C. Membranes were washed 4 x 10 min in TBST and blotted with secondary antibodies (Horse anti-Rabbit-HRP, Vector Labs, 1:5000; Horse anti-Mouse-HRP, Vector Labs, 1:5000) for 2 hr at room temperature. The membranes were again washed 4 x 10 min in TBST, incubated in ECL solution (GE Healthcare Life Sciences), and exposed to film. After developing, the films were digitized on a flatbed scanner.

### Enzyme-linked immunosorbent assay (ELISA)

IL6, TNFα and IL-1β level in brain lysates collected from mice at 3 hr and 3 day after TBI were determined using mouse ELISA Ready-SET-Go kit (eBioscience). 100 μl of concentration-determined protein samples were used for detection according to the manufacturer’s instruction. p65 levels in brain nuclear fraction from 2–3 month adult mice were determined using Cayman NFκB ELISA kit according to manufacturer’s instruction. The absorbance was read on a spectrophotometer with a wavelength of 450 nm. Absolute cytokine amount was calculated based on the standard curve. Concentration of ELISA-determined molecules was quantified as absolute amount determined by the kit divided by total protein amount loaded and presented as pg/μg total protein.

### Histology and immunostaining

Mice were deeply anaesthetized, transcardiac perfused with 4% PFA, and post-fixed in 4% PFA overnight at 4°C. Brains were then transferred to water and then dehydrated in progressively increasing concentrations of ethanol before xylene incubation and paraffin embedding. 8 μm coronal sections were cut on a microtome, dewaxed in xylene and then rehydrated in progressively decreasing concentrations of ethanol. For Nissl staining, sections were incubated in cresyl violet solution and then rinsed in water before dehydration and coverslip application with a permanent mounting medium.

For immunostaining, antigen retrieval was performed by boiling the sections in citrate buffer solution. Sections were then permeabilized in PBS with 0.1% Triton X-100 (PBST) and blocked with 3% goat serum in PBST for 1 hr before incubation with primary antibodies (Rabbit anti-GFAP, Sigma,1:1000; Rabbit anti-Iba1, Wako,1:1000) diluted in blocking solution overnight at 4°C. Sections were then washed in PBST 5 x 3 min before incubation with secondary antibodies (Goat anti-Rabbit-Alexa555, Invitrogen, 1:2000; Goat anti-Mouse-Alexa488, Invitrogen, 1:2000) diluted in blocking solution for 2 hr at room temperature. Sections were again washed in PBST 5 x 3 min and placed on coverslips with Prolong Gold AntiFade Reagent with DAPI (Invitrogen). Images were taken using a Nikon epifluorescent microscope using Metamorph software or with a high resolution slide scanner.

### Quantification of pyramidal neuronal number in CA1 region and gliosis

3–4 equivalent brain sections were chosen from each mouse for each type of quantification using dentate gyrus as a reference. The total pyramidal neuronal number in the sampling regions with a length of 400 μm was counted blindly using Nissl-stained sections. For quantification of gliosis, The total number of GFAP- or Iba1-positive cells in dentate gyrus and the parallel cortical regions were counted per high power field. Each group was represented by 3–4 mice.

### Quantification of relative ventricular size

Five to 6 equivalent Nissl-stained brain sections per genotype from mice survived 14 days after TBI were selected based on the morphology of dentate gyrus in the uninjured side for quantification of ventricular size following CCI. Adobe Photoshop was used to quantify pixel number of the ventricle area and whole brain substances. Relative ventricular sizes were calculated as ventricular pixel number divided by pixels of brain substances.

### Statistics

All data are presented as mean ± SEM. Outliers were identified using Grubbs’ method with α = 0.05. Pairwise comparisons were analyzed using a two-tailed Student’s *t*-test, while a one-way or two-way ANOVA followed by Bonferroni post-hoc analysis was used for multiple comparisons. P values less than or equal to 0.05 were considered statistically significant.

## Competing interests

The authors declare they have no competing interests.

## Author's contributions

HL carried out the biochemical and immunohistochemical experiments, analyzed data and contribute to manuscript drafting. DJS initiated and designed the study, generated the mice, collected and analyzed molecular and MRI data. SSKJ, JR and CSR contributed to the TBI execution. BRB and RGP contributed to the MRI scan. HZ provided critical feedback on experimental design and approach, data analysis and wrote the manuscript. All authors read and approved the final manuscript.
